# Pulmonary echinococcosis mimicking tuberculosis in a child from a dual-endemic region: a case report

**DOI:** 10.3389/fped.2025.1562829

**Published:** 2025-04-07

**Authors:** Yiyuan Li, Yang Liu, Qin Guo

**Affiliations:** ^1^ Department of Pediatrics, West China Second Hospital, Sichuan University, Chengdu, Sichuan, China; ^2^ Key Laboratory of Birth Defects and Related Diseases of Women and Children, Ministry of Education, Chengdu, Sichuan, China

**Keywords:** pulmonary echinococcosis, pulmonary tuberculosis, children, pulmonary cysts, surgery

## Abstract

**Background:**

Pulmonary echinococcosis represents a significant health challenge, particularly in endemic regions, and is associated with substantial morbidity and mortality. Its nonspecific clinical presentation and radiological diversity often lead to misdiagnosis. Here, we report a case of pulmonary echinococcosis initially misdiagnosed as pulmonary tuberculosis.

**Case presentation:**

We report a case of a 13-year-old girl from a region endemic for both echinococcosis and tuberculosis. She initially presented with recurrent cough, hemoptysis, night sweats, and a pulmonary cystic lesion and was diagnosed with pulmonary tuberculosis. However, her condition progressively deteriorated despite antituberculosis therapy. Ultimately, surgical intervention and histopathological examination confirmed pulmonary echinococcosis, and the patient achieved complete recovery after therapy.

**Conclusion:**

For patients from regions endemic for both tuberculosis and echinococcosis who present with cough, hemoptysis, or pulmonary cystic or cavitary lesions, it is crucial to differentiate pulmonary echinococcosis from pulmonary tuberculosis. The final diagnosis should be supported by other microbiological-serological and/or histopathological tests.

## 1 Introduction

Pulmonary echinococcosis, a zoonotic parasitic disease caused by the larval stage of cestodes of the genus *Echinococcus*, poses a significant public health burden, particularly in pastoral regions. It not only leads to economic losses in the livestock industry but also causes severe health complications and even death in humans (1, 2). China has been reported to have the highest number of echinococcosis cases, accounting for 73.55% of all recorded cases worldwide (3). The clinical manifestations of pulmonary cystic echinococcosis depend on the cyst volume. Small pulmonary echinococcosis lesions are often asymptomatic, whereas large cysts can result in acute, life-threatening complications (4, 5). The most common symptoms were cough (53%–62%), chest pain (49%–91%), dyspnea (10%–70%) and hemoptysis (12%–21%), and other less frequently symptoms include dyspnea, malaise, nausea, and vomiting and thoracic deformations (6). Due to its non-specific symptoms and diverse radiological manifestations, pulmonary echinococcosis has a broad differential diagnosis, making its diagnosis challenging and prone to misdiagnosis (7). When a pulmonary cystic lesion ruptures and forms a cavity, it is challenging to distinguish it from pulmonary tuberculosis (6). Here, we present a case of pulmonary echinococcosis in a child who was initially misdiagnosed with pulmonary tuberculosis, aiming to raise awareness of pulmonary echinococcosis and reduce misdiagnosis.

## 2 Case presentation

A 13-year-old female patient from a rural region of Tibet presented with a one-year history of recurrent cough, hemoptysis and night sweats, without fever. She had been diagnosed with pulmonary tuberculosis at a local hospital following chest x-ray and computed tomography (CT) findings. The patient or her family were unable to provide information on whether any microbiological tests for tuberculosis were performed. Following eight months of antituberculosis treatment, her symptoms of cough and hemoptysis partially improved, but she developed exertional dyspnea and failed to gain weight. Four days prior to admission, the patient experienced severe paroxysmal coughing episodes with hemoptysis, shortness of breath, and profuse sweating. Her maternal uncle had a history of tuberculosis during childhood, though they had no prolonged contact. She consumed undercooked beef and lived in an environment where cattle and sheep were raised.

**Figure 1:**
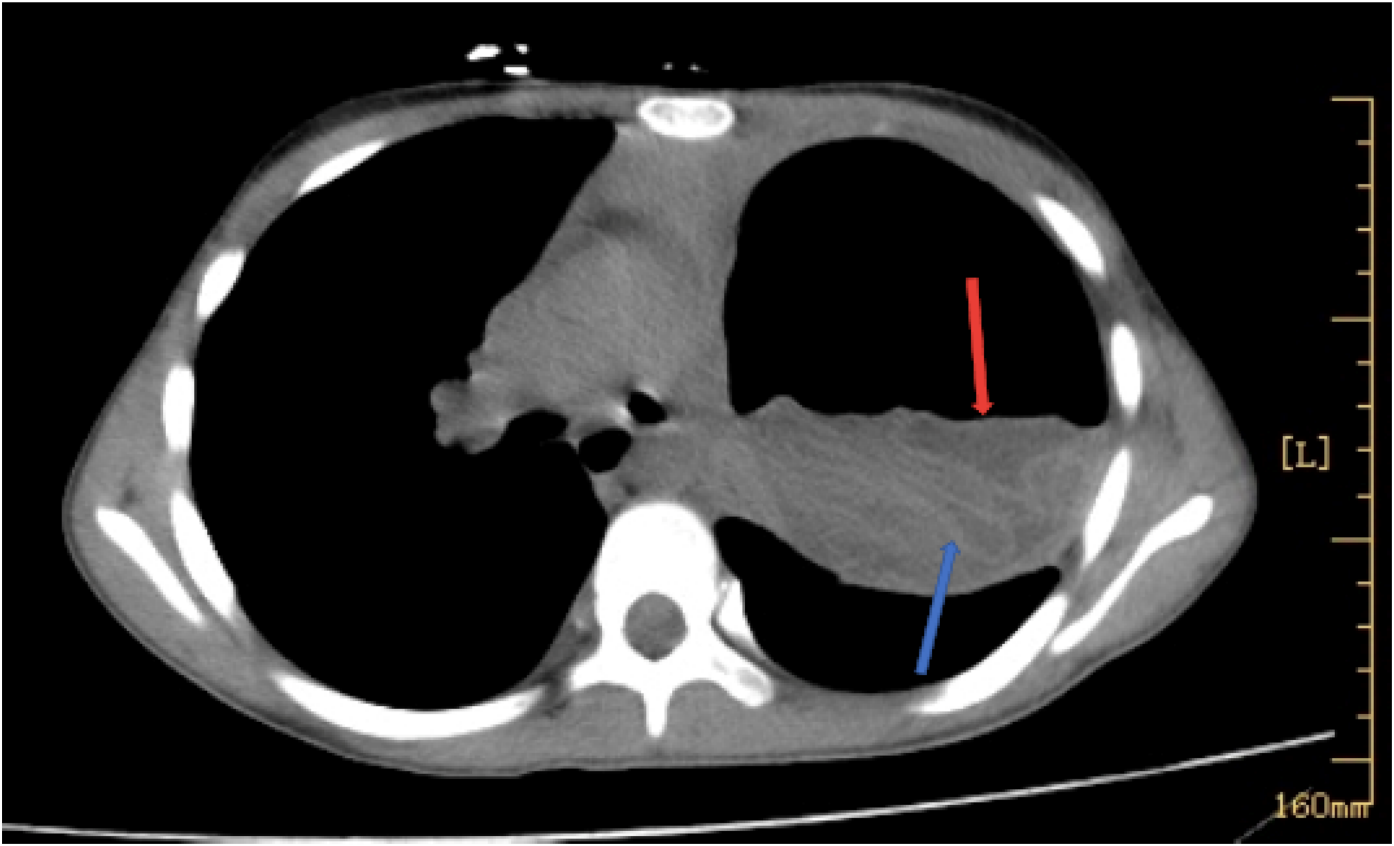
Image of the chest CT. The images revealed cystic liquid shadow (the red arrow), and whirl sign or a ribbon sign (the blue arrow).

On admission, her physical examination revealed: heart rate of 92 beats per min, respiratory rate of 29 breaths per min, and a blood pressure of 93/61 mmHg. The patient appeared emaciated, with restricted left-sided respiratory movement and diminished breath sounds in the left lower lung. Crackles were audible. Heart rhythm was regular, but heart sounds were slightly muffled. No other abnormalities were noted on examination. There was no visible Bacille Calmette-Guérin scar on her arms. Bloodwork showed elevated eosinophils (1.17 × 10⁹/L), and normal white blood cell count (4.07 × 10⁹/L) and C-creative protein. Chest CT revealed a left lung gas-fluid level with pleural effusion, mediastinal shift, and whirl sign or a ribbon sign, suggestive of hydro-pneumothorax (Figure 1). The tests on admission for tuberculosis, including purified protein derivative (PPD) skin test, interferon-gamma release assay (IGRA), and sputum acid-fast bacilli smear, were negative.

**Figure 2:**
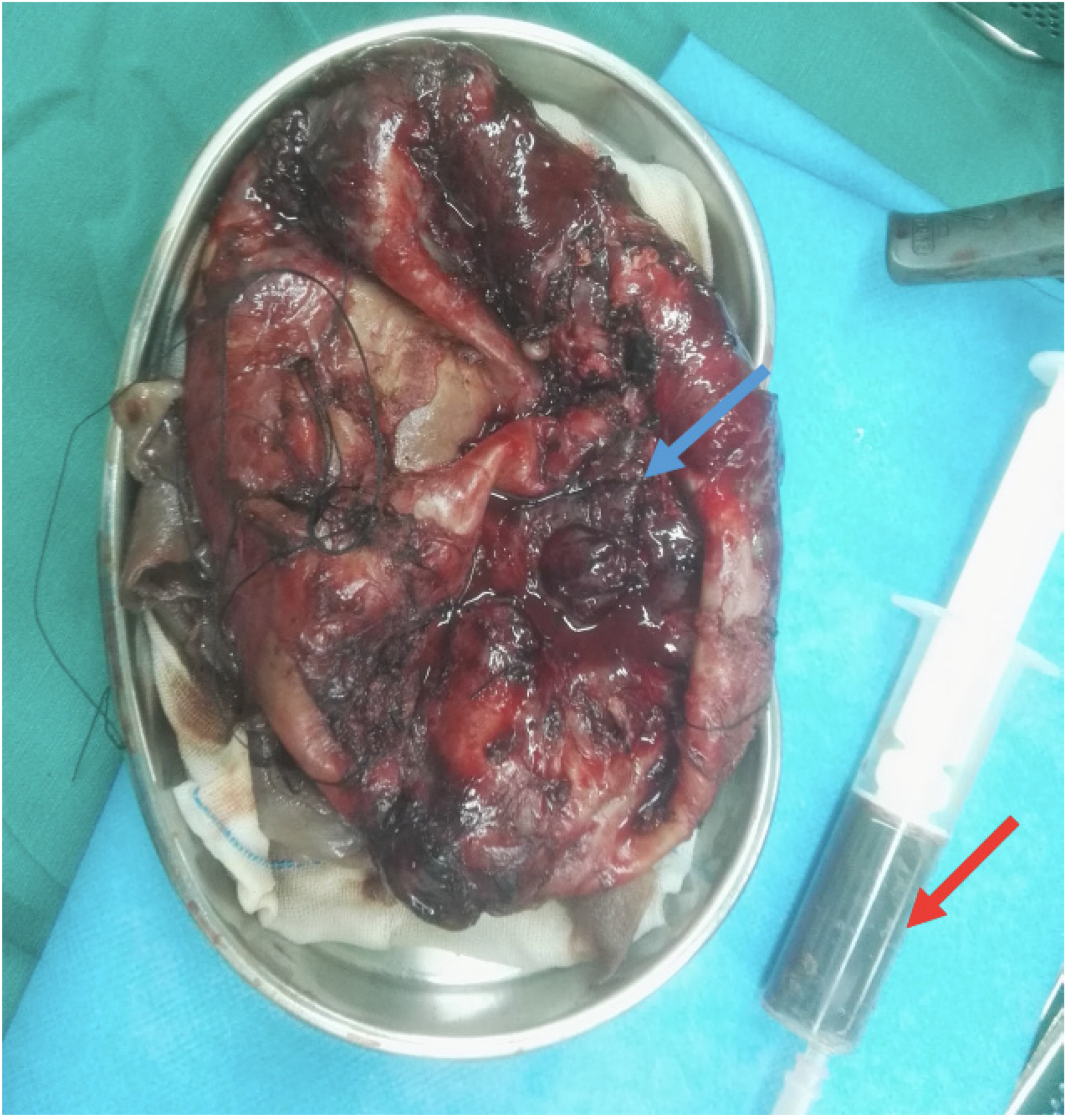
Macroscopic appearance. The left upper lobe exhibits massive cystic changes (the blue arrow), with significant fibrosis and thickening of the cyst wall. The cyst is filled with gas, purulent fluid, and dark brown, granular, melanin-like material (the red arrow).

On the second day of admission, due to progressive respiratory distress, she underwent surgery, during which the lesion and surrounding tissues were completely excised. Intraoperative finding showed that extensive fibrous adhesions on the chest wall and lung surface, with purulent debris and scattered hemorrhagic foci. After removal of the fibrous membrane, a few ruptures were identified, with bubbles emanating from them. The left upper lobe exhibited massive cystic changes, with significant fibrosis and thickening of the cyst wall. The cyst was filled with gas, purulent fluid, and dark brown, granular, melanin-like material, and the inner cyst wall was smooth (Figure 2). The left lower lobe was collapsed due to compression, and there was significant mediastinal shift towards the right side. The microbiological analysis of respiratory secretions yielded negative results for bacterial, fungal, and mycobacterium tuberculosis cultures. Histopathological examination of the pulmonary lesions revealed granulomatous inflammation with the presence of parasitic structures, morphologically consistent with *Echinococcus granuloses* infection (cystic echinococcosis). Postoperative treatment included one month of albendazole, along with symptomatic management, and no complications were observed. Approximately six months post-discharge, the pediatric patient reported no cough, hemoptysis, night sweats or dyspea. Thereafter, the patient did not return for further clinical evaluation or participate in any telephone follow-up assessments.

## 3 Discussion

Hydatid cyst disease, caused by *Echinococcus granulosus,* is a significant public health concern in pastoral regions, where it is endemic (1, 8). The lung is the second most commonly affected organ after the liver (9, 10). The clinical presentation of pulmonary hydatid cysts largely depends on the cyst's size and location. Small, intact cysts are often asymptomatic and may be incidentally detected during imaging studies. In contrast, larger or ruptured cysts can produce significant symptoms such as, cough, hemoptysis, dyspnea and recurrent pneumonia, mimicking pulmonary tuberculosis (4, 11). Additional complications of cyst rupture may include acute respiratory distress syndrome and secondary infections, which can be life-threatening (4). This overlap in symptoms makes distinguishing hydatid cysts from tuberculosis particularly challenging in regions endemic to both disease, as demonstrated in this case. The patient, a 13-year-old girl from a dual-endemic region, presented with recurrent cough, hemoptysis, and night sweats, leading to an initial misdiagnosis of tuberculosis. The temporary improvement of cough and hemoptysis following antituberculosis therapy delayed further investigations, highlighting the diagnostic challenge posed by the nonspecific nature of the symptoms (Table 1). For patients undergoing empirical anti-tuberculosis therapy, if there is no significant clinical improvement after two months, and both acid-fast bacilli smear microscopy and mycobacterium tuberculosis culture yield negative results, it is imperative to re-evaluate the diagnosis of tuberculosis. Particularly in patients with a history of contact with cattle or sheep, the possibility of pulmonary hydatid disease should be strongly suspected and thoroughly investigated.

**Table 1:** Evidence of pulmonary tuberculosis and echinococcosis.

Contents	Pulmonary tuberculosis	Echinococcosis
Recurrent cough	Yes	Yes
Hemoptysis	Yes	Yes
Without fever	Uncertain	Yes
Exposure to TB	Yes	No
Exposure to sheep and cattle	No	Yes
Negative PPD test, IGRA	No	Uncertain
Negative tuberculosis microbiology	No	Uncertain
CT revealing ribbon sign	No	Yes
Granuloma and parasitic body in pathology	No	Yes

Yes, supportive evidence; No, non-supportive evidence; Ucertain, not sure.

Imaging findings are critical for the diagnosis of hydatid cysts. Typical pulmonary hydatid cysts appear on CT scans as well-defined, homogeneous lesions with low density and smooth walls (12). However, rupture can produce characteristic signs such as the air crescent sign, inverse crescent sign, and air bubble sign. Complete rupture may present with imaging findings such as the cumbo sign, whirl sign, waterlily sign, or rising sun sign, which are key indicators for differentiating hydatid cysts from tuberculosis (12, 13). Although imaging plays a central role, overlapping radiological features with tuberculosis, such as cavity formation, may still confound the diagnosis, particularly in complicated cases. However, pulmonary imaging diagnostic technologies are continuously advancing. High-resolution ventilation proton MRI using hyperpolarized propane gas has been demonstrated to achieve both high resolution and ultrafast scan speeds (14, 15). Inhaled diethyl ether, as a gaseous contrast agent, has been validated for its applicability in MRI examinations of a wide range of pulmonary diseases (16). The future application of more advanced imaging technologies may further facilitate the differential diagnosis of diseases.

The presence of pulmonary lesions in a patient with a history of exposure to sheep and cattle, as in this case, should raise suspicion for hydatid cyst disease. The integration of imaging characteristic with serological detection of IgG antibodies against *Echinococcus* can significantly improve diagnostic accuracy. For instance, negative results from both the PPD test and IGRA can effectively rule out tuberculosis (8). Furthermore, direct bronchoscopic visualization combined with biopsy can expedite diagnostic clarification, facilitating timely and effective therapeutic interventions (17). In this case, the negative results for PPD and IGRA tests were useful in revisiting the initial diagnosis of tuberculosis and proceeding with further diagnostic workup.

Surgical intervention is the preferred treatment for pulmonary cystic echinococcosis, particularly in complicated cases involving cyst rupture or secondary infection (18). Complete cyst removal is essential to prevent recurrence and avoid complications such as chronic fistula formation (19). Albendazole therapy is often used postoperatively to prevent recurrence. In this case, postoperative albendazole therapy proved effective, and the patient recovered without recurrence or significant sequelae.

This case highlights several important considerations for clinicians managing patients with pulmonary lesions in endemic regions. Firstly, hydatid cyst disease should always be included in the differential diagnosis for pulmonary cavities, especially in children from dual-endemic regions. Secondly, recognizing typical imaging characteristic and incorporating relevant laboratory tests of PPD, IGRA, and serological detection of IgG antibodies against *Echinococcus* can improve diagnostic accuracy. Thirdly, the clinical symptoms of tuberculosis and pulmonary hydatid disease may overlap, leading to diagnostic confusion. Differential diagnosis should be established through comprehensive evaluation, including microbiological assays for *Mycobacterium tuberculosis*, assessment of prior tuberculosis exposure or animal contact history, serological testing for *Echinococcus-specific* IgG antibodies, analysis of pulmonary imaging features, and, when indicated, histopathological examination of affected tissues. Finally, multidisciplinary management, including timely surgical intervention and postoperative pharmacological therapy, is crucial for ensuring favorable outcomes.

## 4 Conclusion

The clinical presentation of echinococcosis is non-specific, and its CT imaging features are diverse, making the diagnosis of pulmonary echinococcosis challenging. When a patient presents with cough, hemoptysis, dyspnea, and pulmonary cavities, distinguishing it from pulmonary tuberculosis can be difficult. Epidemiological history (such as exposure to tuberculosis, contact with dogs or sheep), PPD skin test, IGRA, tuberculosis microbiological tests, serological testing for *Echinococcus-specific* IgG antibodies, and characteristic imaging signs of echinococcosis, can assist in diagnosis. Surgical resection and histopathological examination are crucial for confirming the diagnosis.

## Data Availability

The original contributions presented in the study are included in the article/Supplementary Material, further inquiries can be directed to the corresponding author.
